# Compound Risks of Hurricane Evacuation Amid the COVID‐19 Pandemic in the United States

**DOI:** 10.1029/2020GH000319

**Published:** 2020-12-01

**Authors:** Sen Pei, Kristina A. Dahl, Teresa K. Yamana, Rachel Licker, Jeffrey Shaman

**Affiliations:** ^1^ Department of Environmental Health Sciences, Mailman School of Public Health Columbia University New York NY USA; ^2^ Climate and Energy Program, Union of Concerned Scientists Oakland CA USA; ^3^ Climate and Energy Program, Union of Concerned Scientists Washington DC USA

**Keywords:** COVID‐19, hurricane, evacuation, climate, extreme events, epidemiology

## Abstract

The 2020 Atlantic hurricane season was extremely active and included, as of early November, six hurricanes that made landfall in the United States during the global coronavirus disease 2019 (COVID‐19) pandemic. Such an event would necessitate a large‐scale evacuation, with implications for the trajectory of the pandemic. Here we model how a hypothetical hurricane evacuation from four counties in southeast Florida would affect COVID‐19 case levels. We find that hurricane evacuation increases the total number of COVID‐19 cases in both origin and destination locations; however, if transmission rates in destination counties can be kept from rising during evacuation, excess evacuation‐induced case numbers can be minimized by directing evacuees to counties experiencing lower COVID‐19 transmission rates. Ultimately, the number of excess COVID‐19 cases produced by the evacuation depends on the ability of destination counties to meet evacuee needs while minimizing virus exposure through public health directives. These results are relevant to disease transmission during evacuations stemming from additional climate‐related hazards such as wildfires and floods.

## Key Points

- Hurricane evacuation increases the total number of COVID‐19 cases in both the origin and destination counties of evacuees
- We can minimize excess COVID‐19 cases by directing evacuees to counties with low COVID‐19 transmission rates
- Minimizing evacuation‐related COVID‐19 cases will also require keeping virus transmission rates low in evacuees' destination counties

## 1 Introduction

The combination of the coronavirus disease 2019 (COVID‐19) pandemic, existing racial and socioeconomic inequalities, and environmental stressors exacerbated by climate change is exposing the many ways in which “compound risks” threaten human lives and well‐being while straining the ability of governments at all scales to limit the damage from any one threat on its own (Phillips et al., 2020). Intersections of climate extremes with the pandemic—recent widespread flooding in South Asia at a time of rapidly increasing COVID‐19 caseloads, for example—have made clear that the consequences of such compound risk events can be lethal, though the underreporting of cases around the world (Lau et al., 2020) and widely varying testing capabilities (Kavanagh et al., 2020) make it difficult to accurately quantify their magnitude.

During the 2020 Atlantic hurricane season, COVID‐19 cases were widespread and abundant in many hurricane‐prone areas of the United States. With six hurricanes making landfall in the nation at the time of this writing, the nation repeatedly experienced the collision of geophysical hazards and the pandemic. This study therefore addresses how decision making around one key aspect of hurricane response—evacuation—could influence the trajectory of the pandemic in the United States and be optimized to limit excess COVID‐19 cases. With future global warming expected to continue the observed trend toward increasingly intense Atlantic hurricanes (Kossin et al., 2020; Seneviratne et al., 2012), understanding how to manage and minimize the impact of the combined risks associated with a major hurricane and a global pandemic could prove critical both later this year and in the long term as the risks of such simultaneous disasters increase around the world.

Efficient, effective evacuations—whether voluntary or mandatory—are a critical component of ensuring public safety in the face of geophysical hazards. The scale of recent evacuations from U.S. Southeast and Gulf Coast states has been large: During Hurricanes Matthew (2016), Irma (2017), and Dorian (2019), for example, roughly 2.5, 6.5, and 1.1 million people, respectively, were under evacuation orders (Han et al., 2019; Roache, 2019; Wong et al., 2018). By changing the distribution of people for days or weeks, a large‐scale evacuation amid a pandemic has the potential to alter the trajectory and geographic distribution of infections. And by temporarily relocating from their own homes into potentially shared living arrangements where levels of social contact are higher, evacuees may experience greater transmission risk both during and after an evacuation.

This analysis evaluates how a large‐scale evacuation of the southeast Florida coast from a hypothetical Category 3 hurricane would affect the total number of COVID‐19 cases and their spatial distribution in evacuees' origin and destination counties. This is accomplished by first building a hypothetical hurricane evacuation scenario from previously published studies of evacuation behavior in the southeast U.S. region. We then use a simple, two‐county infectious disease model to identify the most relevant evacuation and epidemiological characteristics influencing COVID‐19 case counts. The findings from these simulations are used to inform experiments with a larger metapopulation model representing SARS‐CoV‐2 transmission in all 3,142 U.S. counties. The metapopulation model was first described in Pei et al. (2020) and has been used by federal, state, and local health agencies to inform their response to COVID‐19. Here, we adapt this metapopulation model to simulate hurricane evacuation scenarios in order to quantify potential increases in transmission related to an evacuation. We show that the number of excess cases occurring during an evacuation is sensitive to the choice of destination locations for the evacuees. We demonstrate the use of an optimization algorithm to minimize the number of excess cases resulting from an evacuation event by directing evacuees to destinations with low COVID‐19 transmission rates.

## 2 Materials and Methods

### 2.1 Hurricane Evacuation Scenario Development

To develop a hypothetical hurricane evacuation scenario for southeast Florida, we drew from previously published hurricane evacuation studies focused on Category 3+ hurricanes that have affected the Southeast or Gulf Coast regions of the United States (Baker, 1994; Cutter et al., 2011; Lindell et al., 2011, 2020; Martín et al., 2017; Noltenius, 2008; Stein et al., 2010; Wong et al., 2018; Wu et al., 2012; Yin, 2013; Yin et al., 2014; Zhang et al., 2004). We chose southeast Florida for several reasons: It is a region that has experienced numerous hurricanes in the past; evacuations from the region can involve millions of people; and there were previously published data regarding evacuation behavior from the region. In this scenario, we assumed a Category 3 hurricane approaching the southeast Florida coast along a track that would necessitate evacuations from Palm Beach (Palm Beach County EMS, n.d.), Broward (Evacuation zones, routes and shelters, 2016), Miami‐Dade (Miami Dade County, 2020), and Monroe (Florida State Emergency Response Team & South Florida Regional Planning Council, 2019) Counties.

We obtained the population under mandatory evacuation orders in each county from GIS shapefiles of the zones of mandatory evacuation from a Category 3+ hurricane for each county (Florida Hurricane Evacuation Zones [FeatureServer], 2019). The mandatory evacuation zones—and therefore the population living within them—are the same for Category 4 and 5 hurricanes as they are for Category 3 storms. We then calculated the percent of the population living within mandatory evacuation zones that would actually comply with evacuation orders based on a Category 3 or stronger hurricane by averaging the compliance rate from eight regionally relevant studies of evacuation behavior (Baker, 1994; Cutter et al., 2011; Lindell et al., 2011; Martín et al., 2017; Stein et al., 2010; Wong et al., 2018; Yin et al., 2014; Zhang et al., 2004). We found that the average evacuation order compliance rate from these studies was 66%, though the compliance rate tends to increase with storm strength. Thus, if were we to model a Category 4 or 5 hurricane, the evacuee population would likely be larger. Because many people living outside of the mandatory evacuation zones voluntarily choose to evacuate during hurricanes as well, we used the same approach to calculate the percent of the population living outside of mandatory evacuation zones but within the affected counties that would evacuate. Based on four studies of this “shadow evacuation” phenomenon, we determined that an average of 47% of county residents outside mandatory evacuation zones would also evacuate (Cutter et al., 2011; Lindell et al., 2011; Wong, Pel, et al., 2020; Wong et al., 2018; Yin et al., 2014). The mandatory and voluntary evacuees together represent 48% of each origin county's population. We then determined that 19% of these evacuees would relocate within their respective counties based on the average from four evacuation behavior studies (Dash & Morrow, 2000; Dow & Cutter, 2002; Martín et al., 2017; Wong, Pel, et al., 2020; Wong et al., 2018; Wu et al., 2012).

Finally, to determine the destination counties of evacuees from each of the four origin counties, we obtained raw post‐Hurricane Irma survey data (Wong, Pel, et al., 2020; Wong et al., 2018). These data allowed us to identify both the destination counties and the percent of evacuees choosing each destination county. We then apportioned evacuees leaving the four origin counties to each destination county.

### 2.2 Two‐County Model of COVID‐19 Transmission

In order to identify the most sensitive factors driving the COVID‐19 transmission during evacuation, we first ran simulations using a simple two‐county model. This model describes the transmission dynamics of COVID‐19 during a hurricane evacuation from an origin (County 1) to a destination (County 2). Mathematically, the transmission dynamics in the origin and destination are depicted by a susceptible‐exposed‐infected‐recovered (SEIR) model. We simulate the disease transmission as a stochastic Markov process using the following equations:

(1) $$S_{i}\left(t+1\right)=S_{i}\left(t\right)-\frac{\beta_{i}S_{i}\left(t\right)I_{i}^{r}\left(t\right)}{N_{i}}-\frac{\mu \beta_{i}S_{i}\left(t\right)I_{i}^{u}\left(t\right)}{N_{i}},$$

(2) $$E_{i}\left(t+1\right)=E_{i}\left(t\right)+\frac{\beta_{i}S_{i}\left(t\right)I_{i}^{r}\left(t\right)}{N_{i}}+\frac{\mu \beta_{i}S_{i}\left(t\right)I_{i}^{u}\left(t\right)}{N_{i}}-\frac{E_{i}\left(t\right)}{Z},$$

(3) $$I_{i}^{r}\left(t+1\right)=I_{i}^{r}\left(t\right)+\alpha \frac{E_{i}\left(t\right)}{Z}-\frac{I_{i}^{r}\left(t\right)}{D},$$

(4) $$I_{i}^{u}\left(t+1\right)=I_{i}^{u}\left(t\right)+\left(1-\alpha \right)\frac{E_{i}\left(t\right)}{Z}-\frac{I_{i}^{u}\left(t\right)}{D},$$

(5) $$R_{i}\left(t+1\right)=R_{i}\left(t\right)+\frac{I_{i}^{r}\left(t\right)}{D}+\frac{I_{i}^{u}\left(t\right)}{D}.$$

Here *N*_*i*_, *S*_*i*_(*t*), *E*_*i*_(*t*), $I_{i}^{r}\left(t\right)$, $I_{i}^{u}\left(t\right)$, and *R*_*i*_(*t*) are the total, susceptible, exposed, reported infected, unreported infected, and recovered population in county *i* on day *t*; *β*_*i*_ is the transmission rate in county *i*; *μ* is the relative transmissibility for unreported infections; *Z* is the average latency period; *D* is the average duration of infectiousness; and *α* is the fraction of reported infections. The effective reproductive number, which quantifies the local transmission rate, is computed as *R*_*e*_ = *β*_*i*_*D*[*α* + *μ*(1 − *α*)]*S*_*i*_/*N*_*i*_ using the next generation matrix approach.

During evacuation, we assume a fraction (*p*_*eva*_) of the population is evacuated from County 1 to County 2 and mixes with the local population for *T*_*eva*_ days. Individuals within each compartment are randomly drawn from the population in the origin. We track the infections in the evacuated population in County 2, which then return to the origin after the evacuation and mix with the population therein. To account for the increased human interactions associated with evacuating to shared living spaces (Wong et al., 2018), we additionally assume the transmission rates in the origin and destination are elevated during a period that spans the evacuation process. Specifically, the transmission rate in the origin is increased by 20% starting from 3 days prior to the evacuation until 3 days after the return of evacuees. The transmission rate in the destination is increased by 20% during the evacuation.

In model simulations, we fixed the following parameters in Equations 1–5: total population *N*_1_ = *N*_2_ = 10^6^; reporting rate *α* = 0.1; relative transmissibility *μ* = 0.64; latency period *Z* = 4 days; and infectious period *D* = 4 days. Denote the daily reported cases in the origin and destination as *case*_*i*_. To initiate model simulations, we set $I_{i}^{r}\left(0\right)=\mathrm{cas}e_{i}D$, $I_{i}^{u}\left(0\right)=I_{i}^{r}\left(0\right)/\alpha -I_{i}^{r}\left(0\right)$, $E_{i}\left(0\right)=I_{i}^{r}\left(0\right)+I_{i}^{u}\left(0\right)$, *R*_*i*_(0) = 0.05*N*_*i*_ and $S_{i}\left(0\right)=N_{i}-E_{i}\left(0\right)-I_{i}^{r}\left(0\right)-I_{i}^{u}\left(0\right)-R_{i}\left(0\right)$. Model simulations were generated for the following stages after Day 0: 14 days of local transmission, 3 days of preevacuation (with elevated transmission rate in the origin), *T*_*eva*_ days of evacuation (with elevated transmission rates in both the origin and destination counties), 3 days of postevacuation (with elevated transmission rate in the origin), and 28 days of additional postevacuation simulation.

The objective of this two‐county modeling exercise was to explore model parameter space and determine which combinations of parameters would produce the most favorable outcomes. We varied six parameters to generate a large number of parameter combinations for use in model simulation: *β*_1_, *β*_2_ = 0.1, 0.2, …, 0.8; *T*_*eva*_ = 3, 4, …, 10 days; *p*_*eva*_ = 0.1, 0.2, …, 0.8; *case*_1_, *case*_2_ = 50, 100, …, 400. In total, 8^6^ = 262,144 parameter combinations were simulated. For comparison, we also ran a simulation without evacuation for each parameter combination and computed the percentage change of total cases in the origin and destination counties attributed to evacuation. We defined favorable parameter combinations as the 10% of simulations producing the lowest percentage increase (or highest percentage reduction) of reported cases in the origin county, the destination county and both counties combined. Within this favorable subset, we further explored the sensitivity of reported COVID‐19 cases to each parameter by inspecting the marginal distributions of those six parameters.

### 2.3 Simulating COVID‐19 Transmission in the United States

Following the two‐county model analysis, we conducted in‐depth COVID‐19 simulations using a nation‐wide metapopulation SEIR model representing all 3,142 U.S. counties (Pei et al., 2020). In this model, disease transmission in each county follows SEIR dynamics but is also influenced by movement to and from other counties. The model equations are presented in the supporting information. Similar models have been used to simulate COVID‐19 transmission in China (Li et al., 2020) and influenza transmission in the United States (Pei et al., 2018; Pei & Shaman, 2020). The local effective reproductive number is derived as *R*_*e*_ = *β*_*i*_*D*[*α* + *μ*(1 − *α*)]*S*_*i*_/*N*_*i*_. To account for reporting delays in COVID‐19 case and death observations, we mapped simulated documented infections to confirmed cases using a separate observational delay model fitted to the U.S. case data (Pei & Shaman, 2020).

We considered two types of movement in the baseline metapopulation model: daily work commuting and random movement. To simulate movement prior to 15 March 2020, we used information on county‐to‐county work commuting that is publicly available from the U.S. Census Bureau. After 15 March, the census survey data are no longer representative due to changes of mobility behavior in response to COVID‐19 control measures. Therefore, in simulating movement after 15 March 2020, we used estimates of the reduction of intercounty visitors to points of interest (POIs) (e.g., restaurants and stores) to inform declines of intercounty movement on a county‐by‐county basis. We generated these estimates using data from SafeGraph (SafeGraph, n.d.). We further assumed that the number of random visitors between two counties is proportional to the average number of commuters between them. As the population present in each county is different during daytime and nighttime, we modeled the transmission dynamics of COVID‐19 separately for these two time periods.

We calibrated the transmission model against county‐level case and death data reported from 21 February through 23 July 2020 (USAFacts, 2020), which produced an estimate of model parameters and state variables. We then ran simulations representing the following stages: 3 days of preevacuation, 7 days of evacuation, 3 days of postevacuation, and 14 days after postevacuation. For comparison, we also ran simulations without evacuation and increase of transmission rates in origin and destination counties. An ensemble of 100 trajectories were generated to represent the uncertainty arising from different initial conditions and stochastic dynamics.

In the modeled hurricane evacuation, *V*_*ji*_ evacuees travel from origin *i* to destination *j* and mix with the local population for *T*_*eva*_ = 7 days, before returning to origin *i*. As for the two‐county model, we increased the transmission rate in origin counties by 20% during the 3‐day preevacuation, 7‐day evacuation, and 3‐day postevacuation. Three scenarios in which the transmission rate in destination counties is increased by 0%, 10%, and 20% were simulated to compare different effects of hosting evacuees on local disease transmission. The parameters and settings in the full model simulation and the following optimization analysis are reported in Table S1.

### 2.4 The Greedy Algorithm to Optimize Evacuation

We developed a greedy optimization algorithm aimed at minimizing total excess COVID‐19 cases by strategically assigning evacuees to optimal destination counties. In the evacuation optimization, we assume that a fraction *p* of evacuees from an origin to a destination will not change their evacuation plans (for reasons such as personal connections at the destination or budgetary limitations) and the capacity of accepting evacuees for each destination *j* is *C*_*j*_. Denote the baseline evacuation matrix as ***V***, where *V*_*ji*_ represents the number of evacuees from origin *i* to destination *j*. The optimization objective is to assign the rest of evacuees (i.e., (1 − *p*) × ∑_*j*_*V*_*ji*_ from origin *i*) to destinations in an optimal way that minimizes the total infections in both origin and destination counties. Finding the exact solution to this combinatorial optimization problem is computationally challenging due to the large number of options.

In this study, we use a practically feasible greedy optimization approach that prioritizes moving the unassigned evacuees to destinations with low *R*_*e*_. Specifically, we start from the evacuation matrix *p****V*** that represents evacuees assigned a destination. In each step of greedy search, we run a series of simulations, each one filling the available evacuee slots in the destination with the lowest *R*_*e*_ from one of the origin counties. We select the origin county that generates the minimum number of reported cases and assign them to the destination county. We repeat this greedy search for each successive destination county until all evacuees are assigned a destination. The pseudocode for this greedy algorithm is provided in the supporting information. In this study, we assume 10% of evacuees from an origin to a destination in the baseline evacuation matrix ***V*** cannot be reallocated (i.e., *p* = 0.1), and the capacity of each destination is 120% of the evacuees in the baseline scenario ***V*** (i.e., *C*_*j*_ = 1.2∑_*i*_*V*_*ji*_).

## 3 Data

The model optimization uses COVID‐19 county‐level confirmed case and death data from USAFacts (USAFacts, 2020). These data are compiled by USAFacts from Centers for Disease Control and Prevention (CDC), state and local health departments. County population data are from the U.S. Census Bureau (U.S. Census Bureau, 2015).

Movement between counties in the metapopulation model is derived from data documenting county‐to‐county work commuting that are publicly available from the U.S. Census Bureau (U.S. Census Bureau, 2015) and POI data from SafeGraph (SafeGraph, n.d.).

Data used to construct the hurricane evacuation scenario are publicly available in references cited throughout section 2. The code and resulting data sets are available with no restrictions in Pei (2020).

## 4 Results

### 4.1 Identifying Key Parameters Using a Two‐County Model

**Figure 1:**
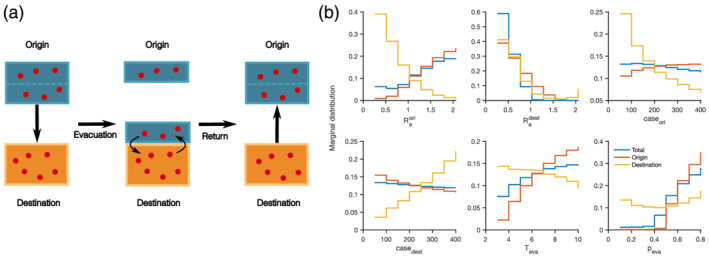
Results from the two‐county model showing that origin and destination transmission rates have the greatest influence on final case numbers. (a) A schematic diagram for the two‐county model. Blue and orange boxes represent the origin and destination populations. Red dots within boxes represent infected individuals. (b) The marginal distribution of six parameters for the top 10% of combinations that lead to the lowest percentage increase (or highest percentage reduction) of reported cases in the origin county (solid red lines), the destination county (solid orange lines), and both counties combined (solid blue lines). Here $R_{e}^{\mathrm{ori}}$ and $R_{e}^{\text{dest}}$ represent the transmission rates in the origin and destination; *case*_*ori*_ and *case*_*dest*_ represent the daily cases in the origin and destination; *T*_*eva*_ is the duration of evacuation; and *p*_*eva*_ is the fraction of the origin population evacuating. The step changes in (b) are due to the discrete values used in model simulations.

We used a simplified, two‐county metapopulation model, representing a generic pair of origin and destination counties, to determine the factors that have greatest influence on COVID‐19 case numbers (section 2 and Figure 1a). Specifically, we evaluated the effects of six evacuation and epidemiological characteristics on COVID‐19 case numbers: transmission rates in origin and destination counties (quantified by the effective reproductive number, *R*_*e*_), the fraction of the origin county population that evacuates (*p*_*eva*_), the duration of the evacuation period (*T*_*eva*_), and daily case numbers in the origin and destination counties (*case*_*ori*_ and *case*_*dest*_). We simulated an evacuation by moving a fraction of the population from the origin to the destination county. Evacuees then mixed with the population of the destination county, before returning home. This simulation was repeated using different combinations of each of the six characteristics in order to determine the effects of each on COVID‐19 case numbers during and following the evacuation.

We found that transmission rates in the origin and destination counties were the primary determinant of case numbers (section 2 and Figure 1b): evacuating individuals from a high‐*R*_*e*_ origin to a low‐*R*_*e*_ destination produced fewer additional cases in the origin county and in the origin and destination county combined. For the destination county alone, it was preferable to accept evacuees from a low‐*R*_*e*_ origin. However, in a real hurricane landing, the counties that require evacuation are determined by the path of the hurricane; that is, a low‐*R*_*e*_ origin cannot be stipulated. The length of evacuation and the number of people evacuating also influenced case numbers; however, these two characteristics are also expected to be shaped more by the specific circumstances necessitated by a particular hurricane rather than by public health directives.

### 4.2 Full Model Simulations of Hurricane Evacuation Scenarios

Next we used a national county‐scale metapopulation model and a suite of scenarios to further explore how transmission rates and hurricane evacuation affect COVID‐19 incidence in origin and destination counties (section 2). All scenarios assume that a Category 3 hurricane is approaching southeast Florida and that people living in evacuation zones within Palm Beach, Broward, Miami‐Dade, and Monroe Counties are ordered to evacuate. Based on studies of evacuation compliance and behavior in this region for Category 3+ hurricanes, we estimate that 48% of each county's population would evacuate (Baker, 1994; Cutter et al., 2011; Lindell et al., 2011; Martín et al., 2017; Stein et al., 2010; Yin et al., 2014; Wong, Pel, et al., 2020; Wong et al., 2018; Zhang et al., 2004) (section 2). Assuming that 19% of evacuees relocate elsewhere within their respective counties, this leads to a total of 2.3 million evacuees leaving the four affected counties (Dash & Morrow, 2000; Dow & Cutter, 2002; Martín et al., 2017; Wong, Pel, et al., 2020; Wong et al., 2018; Wu et al., 2012).

**Figure 2:**
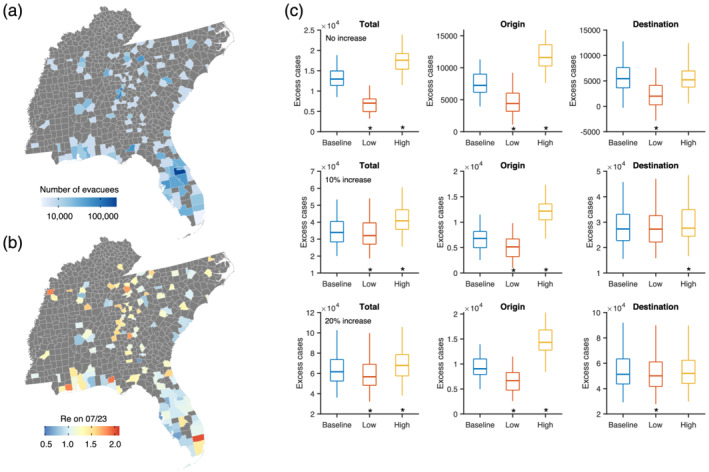
Simulations for evacuation using the national county‐level transmission model. (a) The number of evacuees accepted by destination counties in the baseline scenario in southeast United States. (b) The estimated effective reproductive numbers *R*_*e*_ for both origin and destination counties in southeast United States on 23 July 2020. (c) Comparison of excess cases in origin and destination counties combined (left column), only origin counties (middle column) and only destination counties (right column) for the baseline, low and high evacuation scenarios. Simulations were performed for three settings: no increase (top row), 10% increase (middle row), and 20% increase (bottom row) of the transmission rates in destination counties. Box plots show the median and interquartile and whiskers show the 95% CIs. Asterisks indicate that excess cases are significantly lower or higher than the baseline scenario (Wilcoxon signed rank test, *p* < 10^−5^). Results are obtained from 100 model simulations; the box and whisker distributions show variations across simulation runs.

For each scenario, evacuees were assigned to a different set of destination counties based on a list of 165 possible destinations across 26 states identified during post‐Hurricane Irma surveys (Wong, Pel, et al., 2020; Wong et al., 2018). In the baseline scenario, evacuees were assigned to all 165 destination counties in proportion to observed evacuation choices during Hurricane Irma. The number of evacuees assigned to each destination county in the southeast United States (Alabama, Florida, Georgia, Kentucky, Mississippi, North Carolina, South Carolina, and Tennessee) for this baseline scenario is shown in Figure 2a (see full map in Figure S1a). In order to explore the effects of destination county transmission levels on the number of evacuation‐associated COVID‐19 cases, we further proposed two hypothetical scenarios in which evacuees were assigned to locations with high *R*_*e*_ or low *R*_*e*_, as estimated at the start of the evacuation scenario. In the high (low) *R*_*e*_ scenario, 90% of the evacuees assigned to each county in the baseline scenario were instead diverted to the subset of 82 counties with the highest (lowest) *R*_*e*_, weighted by the proportion of evacuees sent to each of these counties in the baseline scenario (section 2).

Scenario projections were initiated from the model state calibrated to observed county‐level COVID‐19 case and death data from 21 February through 23 July 2020 (section 2). The estimated effective reproductive numbers (*R*_*e*_) in origin and destination counties at the start of simulations are shown in Figure 2b (see full map in Figure S1b). Evacuees tend to stay with friends or family, in hotels/motels, or in public shelters, each of which would likely increase transmission opportunities relative to simply staying home (Wong et al., 2018). To reflect this, we assume the COVID‐19 transmission rate in destination counties increases during the evacuation period by either 0%, 10%, or 20%. These different levels of transmission rate can be interpreted as varying levels of control effected in destinations during evacuation, as well as differences in transmission potential associated with different types of accommodation (e.g., staying with friends/families, hotels, or shelters), though these detailed processes are not explicitly simulated. In addition, to reflect periods of hurricane preparation and recovery (Lindell et al., 2020; Noltenius, 2008; Yin, 2013), we elevated the transmission rate in the origin counties by 20% beginning 3 days prior to evacuation and ending 3 days after the return of evacuees (more detailed simulation settings are provided in section 2). For comparison, we also generated simulations for the same period but without evacuation.

In all scenarios, combined cases in origins and destinations are primarily driven by ongoing local COVID‐19 transmission dynamics (Figure S2 and Table S2); however, evacuation does alter disease outcomes. In the baseline scenario, total COVID‐19 cases in the origin and destination counties increase significantly relative to the no‐evacuation scenario (Figure 2c and Table 1; Wilcoxon signed rank test), indicating evacuation in and of itself can cause a statistically significant increase of COVID‐19 cases. Due to the lag between infection and confirmation, a proportion of evacuees who contracted COVID‐19 while in a destination county would be reported in their origin county after returning home. In addition, we assume an elevated transmission rate in origin counties throughout the simulation period to reflect disruptions related to the hurricane (e.g., crowding in grocery and hardware stores as people stock up on supplies) which contributes to the increase in COVID‐19 cases in origin counties. Time series of confirmed cases in both origin and destination counties under the no‐evacuation and high evacuation scenarios are presented in Figure S3.

**Table 1:** Full Metapopulation Model Simulation of the Median Number of Excess Cases in Origin and Destination Counties for Different Evacuation Scenarios (Baseline, Low, High, and Optimized) and Different Increases of Transmission Rates (*R*_*e*_) in Destination Counties (No Change, 10% Increase, and 20% Increase)

	0% *R*_*e*_increase in destination		10% *R*_*e*_increase in destination		20% *R*_*e*_increase in destination	
	Origin excess cases	Destination excess cases	Origin excess cases	Destination excess cases	Origin excess cases	Destination excess cases
Baseline evacuation	7,244 (4.2%)	5,448 (1.0%)	7,853 (4.6%)	28,661 (5.5%)	8,973 (5.2%)	52,478 (10.0%)
High *R*_*e*_ evacuation	11,593 (6.7%)	5,209 (1.0%)	12,173 (7.1%)	27,649 (5.3%)	14,338 (8.3%)	51,996 (9.9%)
Low *R*_*e*_ evacuation	4,409 (2.6%)	1,999 (0.38%)	5,143 (3.0%)	27,270 (5.2%)	6,669 (3.9%)	50,080 (9.5%)
Optimized evacuation	5,441 (3.2%)	3,628 (0.69%)	4,989 (2.9%)	25,919 (4.9%)	7,333 (4.3%)	50,724 (9.7%)

*Note*. Simulations were generated from 24 July to 20 August 2020, representing the following stages: 3 days of preevacuation, 7 days of evacuation, 3 days of postevacuation, and 14 days after postevacuation. Note that the high and low *R*_*e*_ scenarios are not subject to the constraint of destination capacity, whereas the optimized scenario takes into account a hypothetical capacity for each destination county. Percentage increase relative to the no evacuation scenario is also reported in parentheses.

As indicated by the low and high scenarios, the number of COVID‐19 cases resulting from evacuation is significantly lower (higher) than the baseline scenario if evacuees are directed to counties with lower (higher) transmission rates. However, as the transmission rate in the destination counties increases, the differences between the low and high scenarios become less pronounced. This result indicates that the benefits of a directed evacuation would be amplified by more stringent control efforts in destination counties.

### 4.3 Greedy Optimization Method to Minimize COVID‐19 Cases

The model simulations for our hypothetical evacuation scenarios indicate that a strategic evacuation plan could reduce excess COVID‐19 infections. However, these scenarios neither accounted for the accommodation capacities of destination counties nor provided a framework for optimizing evacuation plans.

To address these issues, we developed a greedy search optimization algorithm aimed at minimizing total excess COVID‐19 cases by strategically assigning evacuees to optimal destination counties. As indicated by the two‐county model simulations, evacuating individuals to destinations with low *R*_*e*_ reduces COVID‐19 transmission. However, given the varying prevalence of infection in origin counties and the nonlinear transmission dynamics, it is not straightforward to determine the optimal number of evacuees from each origin county that should be prioritized and redirected to each of the lowest‐*R*_*e*_ destinations. In conducting the optimization, we imposed the following constraints on human movement: (1) We assumed that a fraction of evacuees cannot be redirected from their baseline destination county, representing individuals whose choice of destination will not be influenced by evacuation directives, perhaps due to financial constraints or preferences to stay with family; and (2) we prescribed a capacity limit on the number of evacuees received by each destination county. The greedy search starts from an evacuation matrix representing the evacuees who cannot be redirected from their destination and then iteratively directs the remaining evacuees to destination counties with lowest *R*_*e*_. In each iteration, the algorithm selects which origin counties will be assigned the evacuee slots available in a destination county. This search is repeated for each successive destination county until all evacuees are assigned a destination (section 2 and supporting information).

**Figure 3:**
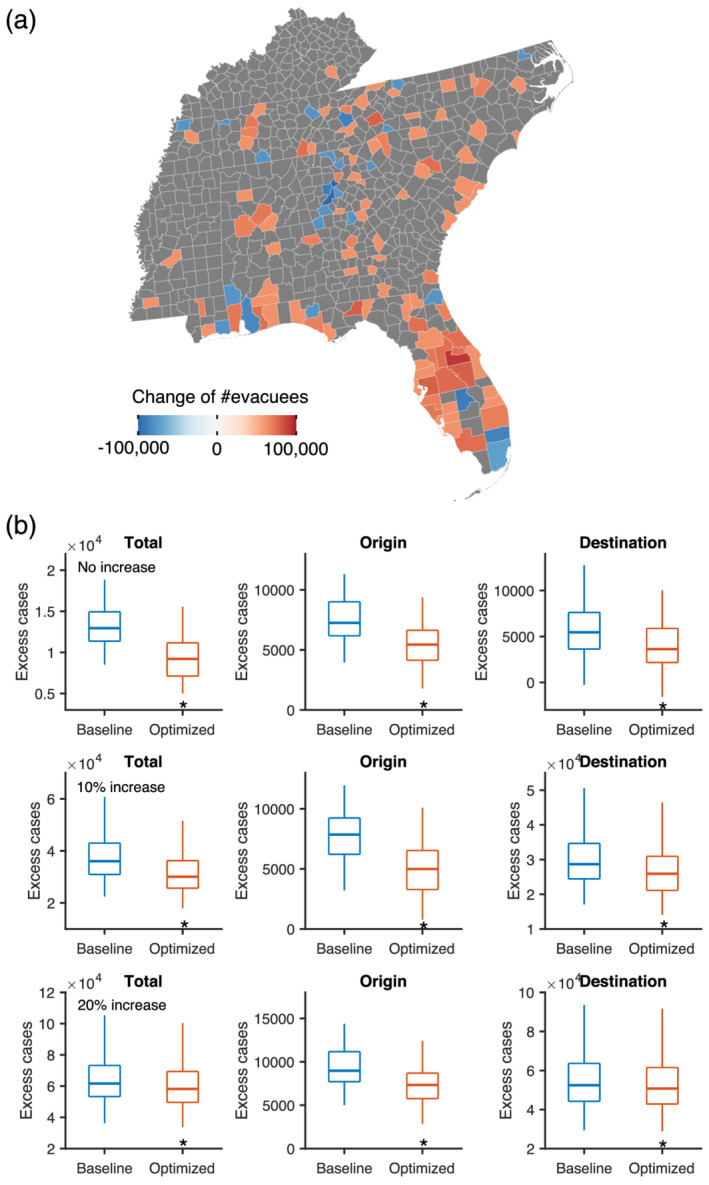
Optimization of evacuation plans. (a) The change in the number of evacuees to destination counties in southeast United States in the optimized evacuation plan compared with the baseline evacuation scenario. Evacuation was optimized for the setting in which transmission rates in destination counties increase by 10%. (b) Excess cases for the baseline and optimized evacuation scenarios are compared for the origin and destination counties combined (left column), only origin counties (middle column), and only destination counties (right column). Simulations were performed for three settings: no increase (top row), 10% increase (middle row), and 20% increase (bottom row) of the transmission rates in destination counties. Boxes and whiskers show the median, interquartile and 95% CIs. Asterisks indicate that excess cases are significantly lower than the baseline scenario (Wilcoxon signed rank test, *p* < 10^−5^). Results are obtained from 100 model simulations.

We repeated this evacuation optimization with three different settings: no increase, 10% increase, and 20% increase of transmission rates in destination counties, again to reflect differences in control efforts and accommodation type. We assumed that 10% of evacuees will maintain their original destination and are thus not redirected and that each destination has a capacity of 120% of the evacuees accepted in the baseline scenario. We then generated the optimized evacuation plan for each setting. During the optimization process, assigning more evacuees to low‐*R*_*e*_ counties led to a reduced number of total infections compared to the baseline scenario (Figure S4). The optimized top 20 destinations for the four origin counties are reported in Tables S3–S5. In Figure 3a, we show the change in the number of evacuees to each destination in southeast United States for the optimized evacuation scenario with a 10% increased transmission rate in destination counties (see full map in Figure S5). In general, evacuees who traveled to high‐*R*_*e*_ destinations in the baseline scenario were redirected to low‐*R*_*e*_ destinations. For all three transmission rate scenarios, the optimized evacuation effectively reduces the number of excess cases in both origin and destination counties compared to the baseline scenario (Figure 3b). The reduction is greatest (up to 30%) for the scenario in which there is no increase in the transmission rate in destination counties, which highlights the crucial role of effective intervention during evacuation. In this optimization example, the fraction of nonallocable evacuees and destination capacity are hypothetical, as these quantities are unknown. If such information were available or could be estimated using socioeconomic and geographic characteristics, the optimization could be tailored to reflect more realistic constraints on evacuation. A sensitivity analysis assuming 20% evacuees cannot be relocated yields similar results (Figure S6).

## 5 Discussion and Conclusions

The results of this study have far‐reaching consequences not only for hurricane evacuation this season but also for long‐term U.S. hurricane preparedness and evacuation planning.

Research suggests that people rely on past experiences when choosing their evacuation routes and destinations (Mesa‐Arango et al., 2013; Wu et al., 2012). This study shows that excess COVID‐19 cases could be minimized by instead directing evacuees to either counties with lower COVID‐19 transmission rates or an optimized set of counties. While decisions about whether to evacuate and where to go ultimately fall to individual households, emergency communications from federal agencies and broadcast meteorologists can influence residents' perceptions of hurricane threats and are seen as trusted sources of information in emergency situations (Lazrus et al., 2012). Because a majority of U.S. residents are concerned about COVID‐19 (Dunn et al., 2020), if the need for a large‐scale evacuation arises, evacuees may turn to these same trusted sources for information on how best to stay safe while evacuating. Local, state, and federal officials who develop evacuation orders and communicate them to the general public may therefore want to consider whether their evacuation‐related communications should include assessments of the relative safety of potential destination counties with respect to COVID‐19 risk rather than allowing default evacuation patterns based on past storms to prevail.

This research shows that the magnitude of the impact of evacuation on COVID‐19 caseloads is highly dependent on conditions in destination counties. The degree to which counties are prepared to host, isolate, and meet the needs of evacuees while also minimizing virus exposure through public health directives such as social distancing and mask wearing will be a key determinant of the impact of evacuation on COVID‐19 case numbers. Preparedness within destination counties is particularly important because, as this analysis shows, destination counties will bear the brunt of the excess COVID‐19 cases that result from an evacuation event. Destination counties must be aware of the influx of evacuees should it occur and must be allocated the financial and human resources needed to ensure the safety of both their residents and the evacuees they are sheltering. Assurances that officials have the necessary resources and procedures in place to keep both the evacuees and the local population safe during the evacuation period could increase the willingness of destination counties with low transmission rates to take in evacuees from origin counties with higher transmission rates.

The U.S. response to the COVID‐19 pandemic has varied widely from state to state and from county to county. As a result of policy, communication, and ideological differences, compliance with mask wearing, for example, has varied substantially even within a given state (Katz et al., 2020). This variability could extend into county‐level hurricane preparedness measures, particularly given that guidance may be issued at the state level while implementation of specific measures is left to counties, as is the case for Florida's current coresponse guidance on hurricane evacuation and COVID‐19 (Florida Division of Emergency Management, 2020).

Centuries of systemic racism in the United States have left Black, Native American, Latinx, and other non‐White people with both higher exposure to and fewer resources to cope with environmental or health‐related stressors compared with White populations (Bell & Ebisu, 2012; Singh et al., 2017). For example, recent research suggests that federal financial aid after disasters is not equitably distributed among communities and may even exacerbate income inequality (Emrich et al., 2020; Howell & Elliott, 2019). Low‐income communities and communities of color consequently struggle to prepare in advance of and recover in the wake of disasters (Baker, 2011; Cleetus et al., 2015).

Due to systemic health inequities, including higher exposure to air pollution (Bell & Ebisu, 2012) and higher rates of underlying health conditions (Colen et al., 2018), COVID‐19 has also disproportionately affected Black, Native American, and Latinx people in the United States (Larsen et al., 2020). These groups have experienced higher infection rates, poorer health outcomes, and deeper declines in employment during the pandemic (Larsen et al., 2020; Oppel et al., 2020; Wu et al., 2020). The additional risks faced because of COVID‐19 and the financial costs associated with evacuation (Wu et al., 2013) could present additional challenges for these segments of the population during hurricane evacuation or discourage them from evacuating altogether and thus further exacerbate inequitable health outcomes.

This study has used idealized scenarios to model hurricane evacuation patterns. These scenarios cannot fully capture many household‐level choices that could alter levels of social contact—and therefore potential COVID‐19 exposure—during the evacuation period. For example, previous studies of hurricane evacuations along the U.S. Southeast and Gulf Coasts show that evacuees strongly and consistently prefer to stay with friends and family over going to hotels/motels or public shelters (Bian et al., 2019; Lindell et al., 2011; Wong et al., 2018; Wu et al., 2012, 2013; Yin et al., 2014). Levels of social contact and potential virus transmission would likely differ across accommodation types, which implies a level of complexity and spatial heterogeneity that is not possible to incorporate within the model used in this study. Similarly, this study does not consider variable levels of exposure to COVID‐19 based on evacuation transportation mode. While evacuees strongly prefer to travel in their own vehicles (Wong, Pel, et al., 2020), shared modes of transportation such as buses or carpools would increase potential virus transmission and exposure. Recent research suggests that for both transportation and shelter, the sharing economy—Internet‐based transactions via companies like Airbnb that allow for peer‐to‐peer sharing of goods and services—could play a role in providing free or affordable resources to evacuees that would enable evacuees to maintain social distancing (Wong, Walker, et al., 2020).

As a result of the ongoing economic and physical toll of the pandemic, household‐level decision making regarding evacuation may differ from that of past years. There are many sociodemographic factors associated with decision making around evacuation including experience with past hurricanes, length of residence, home ownership, age, income, race, employment status, level of social connectivity, social cues, perceived levels of self‐efficacy and risk, and storm conditions (Collins et al., 2018; Demuth et al., 2016; Huang et al., 2016; Lazo et al., 2015; Metaxa‐Kakavouli et al., 2018). While some of these factors (e.g., gender and race) are unchanged from last year, others (e.g., employment status and income) may be either changed or very much influenced by the current COVID‐19 pandemic. In contrast, the idealized scenarios adopted for this study assume that people will choose destination counties and accommodation types that match past choices.

The movement of people in and out of hurricane‐affected counties does not simply cease after all evacuees have returned to their homes. For instance, communities affected by hurricanes often experience an influx of workers who assist with rebuilding and recovery efforts (Fussell, 2009; Jordan, 2019; Theodore, 2017), which could also influence infection rates in the affected counties long after the evacuation period. Postevacuation movement patterns are beyond the scope of the present study.

Critically, hurricane evacuation is intended to save lives and prevent serious injuries to residents of hurricane‐prone regions. While this study evaluates excess COVID‐19 cases resulting from evacuation, it does not evaluate non‐COVID‐19 related risks to human health and lives in the event that people choose to remain in their homes despite receiving evacuation orders—risks that could increase if people are afraid to evacuate out of concern for contracting COVID‐19. Nor does it address evacuations of hospitals, nursing homes, prisons, or other facilities. It will be critical for emergency managers to factor in these—and other—complexities when developing plans (Wong & Shaheen, 2020).

Finally, the results presented here are based on scenarios that, while plausible, are strictly hypothetical. While the overall notion that distributing evacuees to destination counties with low transmission rates minimizes excess cases should theoretically apply to geographies outside Florida or the United States, additional model simulations of such scenarios should be generated. Moreover, while this study models evacuation specifically from a hurricane, the same notion should apply to evacuations from other geophysical hazards such as wildfires and floods, though evacuations from such hazards tend involve fewer people than hurricane evacuations.

The data presented here show that while a large‐scale hurricane evacuation would increase the total number of COVID‐19 cases in the United States, directing evacuees to plausible destination counties with low COVID‐19 transmission rates would minimize the excess cases induced by the evacuation event. These results have far‐reaching implications for immediate emergency management and communications practices, as well as long‐term disaster preparedness.

Faced with the prospect of tens of thousands of additional cases arising from a hurricane evacuation, states and counties at both ends of evacuation routes must be allocated the necessary financial and human resources required to meet evacuees' needs while also ensuring community safety and health through measures intended to reduce COVID‐19 transmission rates. Further, resource distribution must prioritize the nation's most vulnerable groups.

## Conflict of Interest

J. S. and Columbia University disclose partial ownership of SK Analytics. J. S. also discloses consulting for Merck and BNI.

## Supporting information

Supporting Information S1

## Data Availability

Data sets used to construct the hurricane evacuation scenario are available in references throughout section 2. Data for the scenario modeled here, code, and the resulting data sets are available with no restrictions in https://github.com/SenPei‐CU/Hurricane‐COVID (Pei, 2020).
