# Supplementary material for: Compound Risks of Hurricane Evacuation Amid the COVID‐19 Pandemic in the United States
Source: Geohealth. 2020 Dec 1;4(12):e2020GH000319. doi: 10.1029/2020GH000319 (PMC7704390; doi:10.1029/2020GH000319)
Supplement: Supplementary file 1 — Supporting Information S1 [file GH2-4-e2020GH000319-s001.pdf]

AGU PUBLICATIONS

GeoHealth

Supporting Information for

Compound Risks of Hurricane Evacuation amid the COVID-19 Pandemic in the United States

Sen Pei[1*], Kristina A. Dahl[2*], Teresa K. Yamana[1], Rachel Licker[2], Jeffrey Shaman[1]

[1]Department of Environmental Health Sciences, Mailman School of Public Health, Columbia University, New York, NY 10032, USA

[2]Climate & Energy Program, Union of Concerned Scientists, Cambridge, MA, Washington, DC, and Oakland, CA, USA

Contents of this file

Text S1 to S2
Figures S1 to S6
Tables S1 to S5

Introduction

The information here supports the manuscript listed above and includes text, figures, and tables. The data were generated in July and August, 2020.

Text S1. Transmission model for 3,142 US counties

We formulate COVID-19 transmission as a discrete Markov process during both day and night. Daytime transmission lasts for dt_1 days and the nighttime transmission dt_2 days ($dt_1 + dt_2 = 1$). Here, we assume daytime transmission lasts for 8 hours and nighttime transmission lasts for 16 hours, i.e., $dt_1 = 1/3$ day and $dt_2 = 2/3$ day. The transmission dynamics are depicted by the following equations.

Daytime transmission:

$$E_{ij}(t+dt_1) = E_{ij}(t) + \frac{\beta S_{ij}(t)\sum_k I_{ki}^r(t)}{N_i^D(t)}dt_1 + \frac{\mu\beta S_{ij}(t)\sum_k I_{ik}^u(t)}{N_i^D(t)}dt_1 - \frac{E_{ij}(t)}{Z}dt_1 + \theta dt_1 \frac{N_{ij} - I_{ij}^r(t)}{N_i^D(t)}\sum_{k\neq i}\frac{\bar{N}_{ik}\sum_l E_{kl}(t)}{N_k^D(t) - \sum_l I_{lk}^r(t)} - \theta dt_1 \frac{E_{ij}(t)}{N_i^D(t) - \sum_l I_{li}^r(t)}\sum_{k\neq i}\bar{N}_{ki} \quad (1)$$

$$I_{ij}^r(t+dt_1) = I_{ij}^r(t) + \alpha\frac{E_{ij}(t)}{Z}dt_1 - \frac{I_{ij}^r(t)}{D}dt_1 \quad (2)$$

$$I_{ij}^{u}(t+dt_1) = I_{ij}^{u}(t) + (1-\alpha)\frac{E_{ij}(t)}{Z}dt_1 - \frac{I_{ij}^{u}(t)}{D}dt_1 + \theta dt_1 \frac{N_{ij} - I_{ij}^{r}(t)}{N_i^D(t)} \sum_{k\neq i} \frac{\overline{N}_{ik} \sum_l I_{kl}^{u}(t)}{N_k^D(t) - \sum_l I_{lk}^{r}(t)}$$

$$- \theta dt_1 \frac{I_{ij}^{u}(t)}{N_i^D(t) - \sum_l I_{li}^{r}(t)} \sum_{k\neq i} \overline{N}_{ki} \quad (3)$$

$$R_{ij}(t+dt_1) = R_{ij}(t) + \frac{I_{ij}^{r}(t)}{D}dt_1 + \frac{I_{ij}^{u}(t)}{D}dt_1 + \theta dt_1 \frac{N_{ij} - I_{ij}^{r}(t)}{N_i^D(t)} \sum_{k\neq i} \frac{\overline{N}_{ik} \sum_l R_{kl}(t)}{N_k^D(t) - \sum_l I_{lk}^{r}(t)}$$

$$- \theta dt_1 \frac{R_{ij}(t)}{N_i^D(t) - \sum_l I_{li}^{r}(t)} \sum_{k\neq i} \overline{N}_{ki} \quad (4)$$

$$N_i^D(t) = N_{ii} + \sum_{k\neq i} I_{ki}^{r}(t) + \sum_{k\neq i} (N_{ik} - I_{ik}^{r}(t)) \quad (5)$$

Nighttime transmission:

$$E_{ij}(t+1) = E_{ij}(t+dt_1) + \frac{\beta S_{ij}(t+dt_1) \sum_k I_{kj}^{r}(t+dt_1)}{N_j^N} dt_2$$

$$+ \frac{\mu\beta S_{ij}(t+dt_1) \sum_k I_{kj}^{u}(t+dt_1)}{N_j^N} dt_2 - \frac{E_{ij}(t+dt_1)}{Z} dt_2$$

$$+ \theta dt_2 \frac{N_{ij}}{N_j^N} \sum_{k\neq j} \frac{\overline{N}_{jk} \sum_l E_{lk}(t+dt_1)}{N_k^N - \sum_l I_{lk}^{r}(t+dt_1)}$$

$$- \theta dt_2 \frac{E_{ij}(t+dt_1)}{N_j^N - \sum_k I_{kj}^{r}(t+dt_1)} \sum_{k\neq j} \overline{N}_{kj} \quad (6)$$

$$I_{ij}^{r}(t+1) = I_{ij}^{r}(t+dt_1) + \alpha \frac{E_{ij}(t+dt_1)}{Z} dt_2 - \frac{I_{ij}^{r}(t+dt_1)}{D} dt_2 \quad (7)$$

$$I_{ij}^{u}(t+1) = I_{ij}^{u}(t+dt_1) + (1-\alpha) \frac{E_{ij}(t+dt_1)}{Z} dt_2 - \frac{I_{ij}^{u}(t+dt_1)}{D} dt_2$$

$$+ \theta dt_2 \frac{N_{ij}}{N_j^N} \sum_{k\neq j} \frac{\overline{N}_{jk} \sum_l I_{lk}^{u}(t+dt_1)}{N_k^N - \sum_l I_{lk}^{r}(t+dt_1)}$$

$$- \theta dt_2 \frac{I_{ij}^{u}(t+dt_1)}{N_j^N - \sum_k I_{kj}^{r}(t+dt_1)} \sum_{k\neq j} \overline{N}_{kj} \quad (8)$$

$$R_{ij}(t+1) = R_{ij}(t+dt_1) + \frac{I_{ij}^{r}(t+dt_1)}{D} dt_2 + \frac{I_{ij}^{u}(t+dt_1)}{D} dt_2$$

$$+ \theta dt_2 \frac{N_{ij}}{N_j^N} \sum_{k\neq j} \frac{\overline{N}_{jk} \sum_l R_{lk}(t+dt_1)}{N_k^N - \sum_l I_{lk}^{r}(t+dt_1)} - \theta dt_2 \frac{R_{ij}(t+dt_1)}{N_j^N - \sum_k I_{kj}^{r}(t+dt_1)} \sum_{k\neq j} \overline{N}_{kj} \quad (9)$$

$$N_i^N = \sum_k N_{ki} \quad (10)$$

Here, S_{ij}, E_{ij}, I_{ij}^{r}, I_{ij}^{u}, R_{ij} and N_{ij} are the susceptible, exposed, reported infected, unreported infected, recovered and total populations in the subpopulation commuting from county j to county i ($i \leftarrow j$), where $S_{ij} = N_{ij} - E_{ij} - I_{ij}^{r} - I_{ij}^{u} - R_{ij}$; β is the transmission rate of reported

infections; μ is the relative transmissibility of unreported infections; Z is the average latency period (from infection to contagiousness); D is the average duration of contagiousness; α is the fraction of documented infections; θ is a multiplicative factor adjusting random movement; $\bar{N}_{ij} = (N_{ij} + N_{ji})/2$ is the average number of commuters between counties i and j; and N_i^D and N_i^N are the daytime and nighttime populations of county i.

Text S2. The pseudo-code for the greedy optimization algorithm

Input:

Origin $i = 1, 2, \ldots, n$

Destination $j = 1, 2, \ldots, m$, where $R_e(1) \geq R_e(2) \geq \cdots \geq R_e(m)$

Evacuation matrix $\boldsymbol{V} = \{V_{ji}\}$, V_{ji} is the number of evacuees from origin i to destination j in the baseline scenario

Capacity of evacuees that can be accommodated by each destination: C_j

The fraction of evacuees that can't be reallocated for each origin-destination pair: p

Variables:

u_i:: the current number of evacuees in origin i that could be reallocated to different counties

v: the currently available destination county with lowest R_e.

$\boldsymbol{M}$: the current evacuation matrix, M_{vi} is the number of evacuees assigned from origin i to destination v.

Initial conditions:

$v = m$

$u_i = (1-p)\sum_j V_{ji}$

$\boldsymbol{M} = p\boldsymbol{V}$

Algorithm:

```
While max(u_i) > 0
        For i = 1 to n
                M^i = M: reallocating from origin i to v
                M^i_{vi} = min(M_{vi} + (C_v − Σ_j M_{vj}), M_{vi} + u_i)
                u^i_{temp} = u_i − (M^i_{vi} − M_{vi})
                Run projection using M^i
                Inf_i: total infection in all origin and destination counties
        End
        k = min_i Inf_i
        M = M^k
        u_k = u^k_{temp}
        If Σ_j M_{vj} == C_v
                v = v − 1
        End
End
```

Output $\boldsymbol{M}$ as the optimized evacuation matrix.

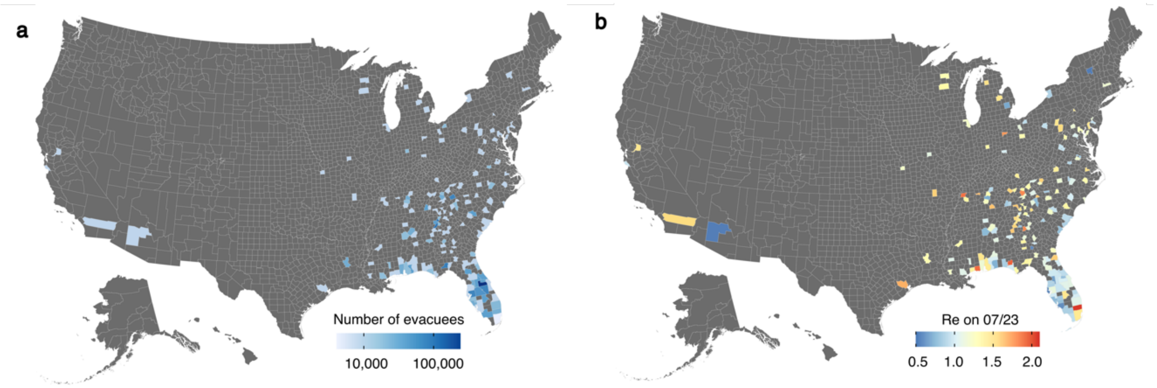

Figure S1. Statistics in destination counties. (a) The number of evacuees accepted by 165 destination counties in the baseline scenario. (b) The estimated effective reproductive numbers, R_e, for both origin and destination counties on July 23, 2020.

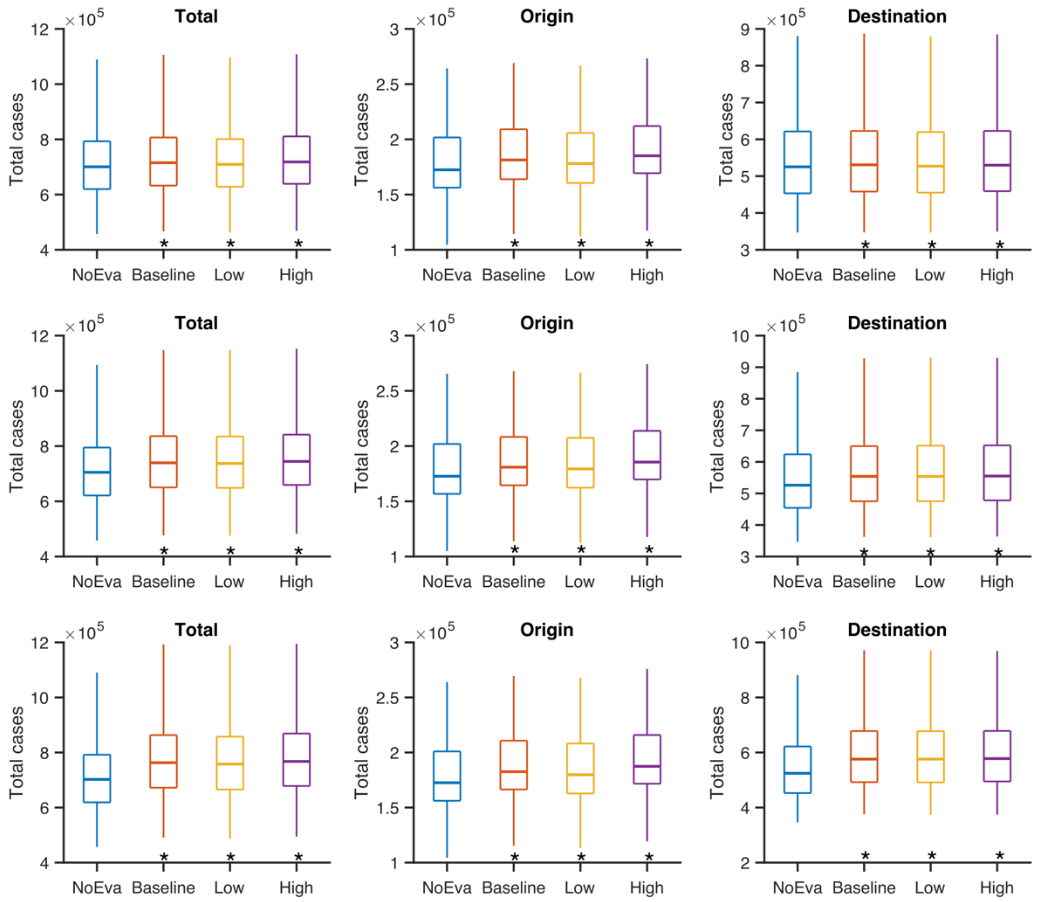

Figure S2. Comparison of total cases in the origin and destination counties combined (left column), the origin counties only (middle column) and destination counties only (right column) for the no-evacuation, baseline, low and high evacuation scenarios. Simulations were performed for three settings: no increase (top row), 10% increase (middle row) and 20% increase (bottom row) of transmission rates in destination counties. Box plots show the median and interquartile and whiskers show the 95% CIs. Asterisks indicate that excess cases are significantly higher than the no-evacuation scenario (Wilcoxon signed rank test, $p < 10^{-5}$).

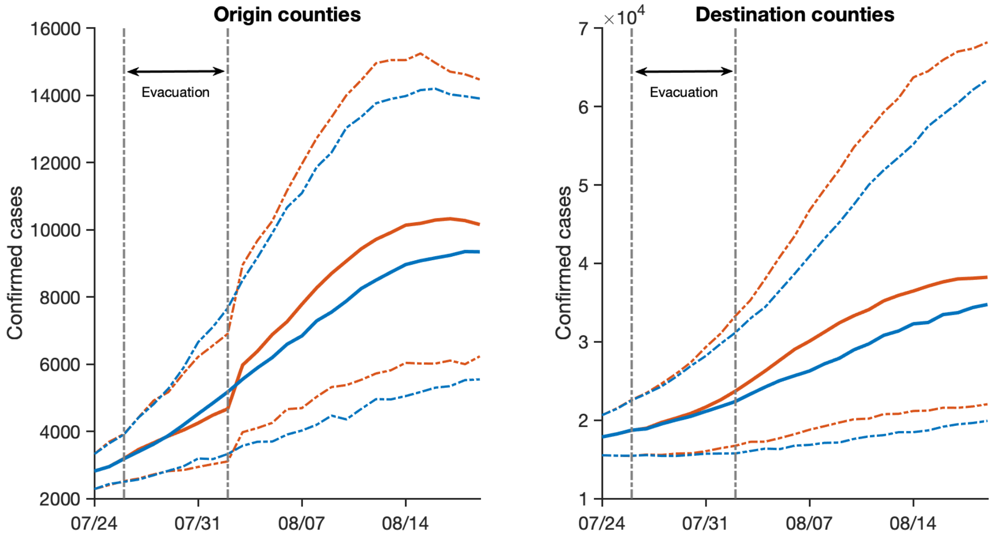

Figure S3. Time series for confirmed cases in origin and destinations counties. Simulations were performed for the no-evacuation scenario (blue line) and the high evacuation scenario with 20% increase of transmission rate in destination counties (red line). Blue and red dashed lines indicate 95% CIs of simulation results from 100 runs.

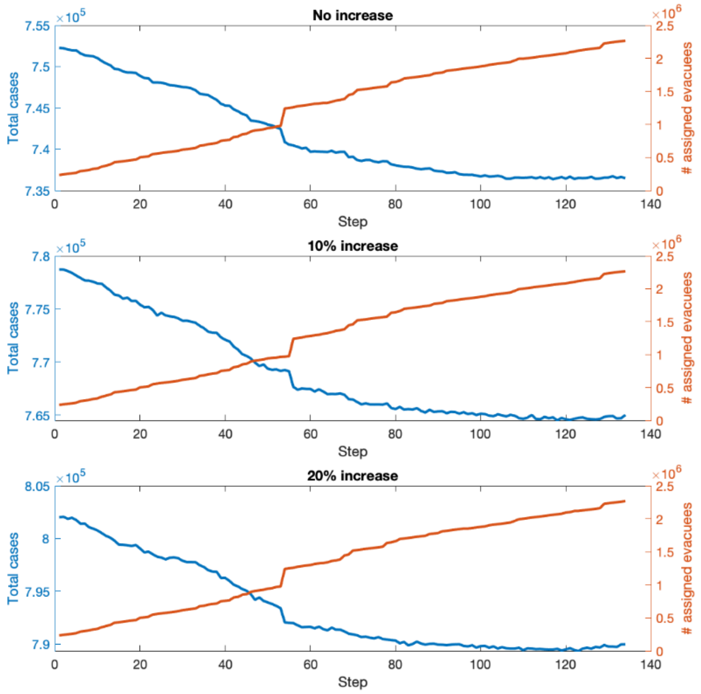

Figure S4. Evolution of the total cases (blue lines) and the number of assigned evacuees (red lines) in the greedy algorithm. Results are shown for the settings with no increase, 10% increase and 20% increase of transmission rates in destination counties. The optimization starts from an evacuation matrix $0.1 \times \boldsymbol{V}$, where $\boldsymbol{V}$ represents the evacuation matrix in the baseline scenario.

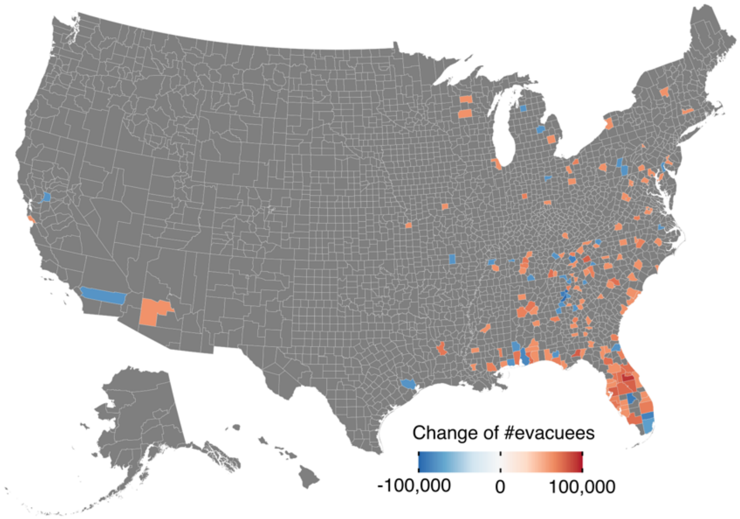

Figure S5. The change in the number of evacuees to destination counties in the optimized evacuation plan compared with the baseline evacuation scenario. Evacuation was optimized for the setting in which transmission rates in destination counties increase by 10%.

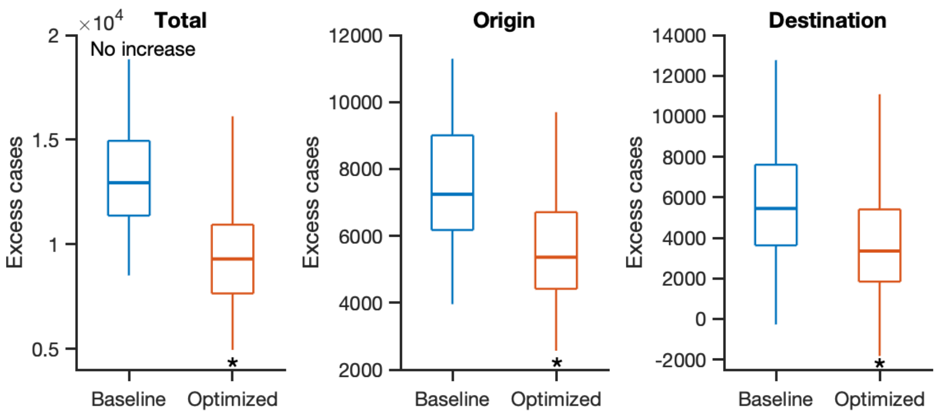

Figure S6. Sensitivity analysis of optimization assuming 20% of evacuees cannot be relocated. Excess cases for the baseline and optimized evacuation scenarios are compared for the origin and destination counties combined (left column), only origin counties (middle column) and only destination counties (right column). Simulations were performed with no increase of transmission rates in destination counties. Boxes and whiskers show the median, interquartile and 95% CIs. Asterisks indicate that excess cases are significantly lower than the baseline scenario (Wilcoxon signed rank test, $p < 10^{-5}$). Results are obtained from 100 model simulations.

Parameter/variable	Values	Source
Baseline transmission rate (β_0, days^{-1})	0.95 (0.84, 1.06)	Pei et al., (2020)
Relative transmission rate (μ)	0.64 (0.56, 0.70)	Pei et al., (2020)
Latency period (Z, days)	3.59 (3.28, 3.99)	Pei et al., (2020)
Infectious period (D, days)	3.56 (3.21, 3.83)	Pei et al., (2020)
Mobility factor (θ)	0.15 (0.12, 0.17)	Pei et al., (2020)
Reporting rate (α)	0.080 (0.069, 0.093)	Pei et al., (2020)
Evacuation time (T_{eva}, days)	7	Assigned
Pre- and post-evacuation (days)	3	Assigned
Increased transmission in origin	20%	Assigned
Increased transmission in destination	0%, 10%, 20%	Assigned
Percentage of evacuees who cannot be reallocated from their baseline destination	10%	Assigned
Capacity of destination counties as a percentage of the number of evacuees received in baseline scenario	120%	Assigned

Table S1. Parameter settings in the full model simulation and optimization. The prior transmission rate in each county is scaled by population density using a baseline transmission rate β_0 as inferred through March 13, 2020. The relative transmission rate (μ), latency period (Z), infectious period (D) and mobility factor (θ) are fixed at posterior values inferred through March 13, 2020. Values are shown for the median and 95% CIs in the parentheses.

	0% R_e increase in destination		10% R_e increase in destination		20% R_e increase in destination	
	**Origin cases**	**Destination cases**	**Origin cases**	**Destination cases**	**Origin cases**	**Destination cases**
**No Evacuation**	172,400	525,320	172,692	526,044	172,562	524,467
**Baseline evacuation**	181,322	530,706	180,901	553,971	182,590	575,891
**High R_e evacuation**	185,100	529,781	185,527	555,062	187,431	577,885
**Low R_e evacuation**	178,029	527,180	179,317	553,875	179,788	576,043

Table S2. The median number of total cases in origin and destination counties for different evacuation scenarios (no evacuation, baseline, low and high) and levels of elevated transmission rates (R_e) in destination counties (no change, 10% increase, 20% increase). In the no evacuation scenario (R_e) in destination counties is not increased. Note that the median excess cases shown in Table 1 is not necessarily the difference of the median total cases between the evacuation and non-evacuation scenarios shown in Table S1. That is, $median(totalcase_{eva}(i) - totalcase_{noeva}(i)) \neq median(totalcase_{eva}(i)) - median(totalcase_{noeva}(i))$, where $totalcase_{eva}(i)$ and $totalcase_{noeva}(i)$ are the total numbers of cases for evacuation and non-evacuation scenarios in the ith simulation.

Palm Beach County FL		Broward County FL		Miami-Dade County FL		Monroe County FL	
Leon County FL	67926	Polk County FL	42726	Orange County FL	277387	Sullivan County TN	8333
Natchitoches Parish LA	25472	Escambia County FL	34181	Hillsborough County FL	60678	Walton County FL	8333
Davidson County TN	25472	Lake County FL	34181	Osceola County FL	60678	Spalding County GA	8333
Marion County IN	16981	Richland County SC	34181	Buncombe County NC	60678	Tift County GA	8333
Jefferson County TN	16981	Volusia County FL	34181	Alachua County FL	34674	Forsyth County GA	649
Camden County GA	16981	Collier County FL	34181	Jefferson County AL	26005	Orange County FL	399
Macon County GA	16981	Hamilton County TN	34181	Mobile County AL	26005	Fulton County GA	125
Forsyth County GA	16357	Seminole County FL	34181	Palm Beach County FL	20372	Leon County FL	100
Montour County PA	8491	Sarasota County FL	25635	St. Tammany Parish LA	17336	Hillsborough County FL	87
Franklin County VA	8491	Sumter County FL	25635	Forrest County MS	17336	Osceola County FL	87
Clay County KY	8491	Lee County AL	17090	Manatee County FL	17336	Buncombe County NC	87
Harrison County MS	8491	St. Johns County FL	17090	Prince Edward County VA	17336	Polk County FL	62
Dougherty County GA	8491	Bay County FL	17090	Oneida County WI	8668	Broward County FL	50
Anderson County TN	8491	Medina County OH	17090	Stanly County NC	8668	Cobb County GA	50
Gibson County TN	8491	Greenville County SC	17090	Baltimore County MD	8668	Highlands County FL	50
Sumter County GA	8491	Tuscaloosa County AL	17090	Watauga County NC	8668	Alachua County FL	50
Florence County SC	8491	Williamson County TN	17090	Winchester city VA	8668	Escambia County FL	50
Marathon County WI	8491	Wake County NC	13191	Maury County TN	8668	Lake County FL	50
Gulf County FL	8491	Montgomery County MD	8545	Queen Anne's County MD	8668	Richland County SC	50
Jefferson County FL	8491	Hamilton County OH	8545	Lexington County SC	8668	Volusia County FL	50

Table S3. The optimized evacuation plan (no increase of transmission rates in destination counties). We show the top 20 destinations for each origin county.

Palm Beach County FL		Broward County FL		Miami-Dade County FL		Monroe County FL	
Leon County FL	67926	Polk County FL	42726	Orange County FL	277387	St. Johns County FL	8969
Jefferson County AL	25472	Escambia County FL	34181	Hillsborough County FL	60678	Charleston County SC	8333
Mobile County AL	25472	Lake County FL	34181	Osceola County FL	60678	Walton County FL	8333
Marion County IN	16981	Richland County SC	34181	Buncombe County NC	60678	Oakland County MI	8333
Macon County GA	16981	Volusia County FL	34181	Alachua County FL	34674	Orange County FL	399
Lee County AL	16981	Collier County FL	34181	Natchitoches Parish LA	26005	Fulton County GA	125
Jefferson County TN	11527	Hamilton County TN	34181	Davidson County TN	26005	Leon County FL	100
Montour County PA	8491	Seminole County FL	34181	Palm Beach County FL	20372	Hillsborough County FL	87
Franklin County VA	8491	Sarasota County FL	25635	Camden County GA	17336	Osceola County FL	87
Clay County KY	8491	Sumter County FL	25635	St. Tammany Parish LA	17336	Buncombe County NC	87
Harrison County MS	8491	Bay County FL	17090	Forrest County MS	17336	Polk County FL	62
Dougherty County GA	8491	Manatee County FL	17090	Forsyth County GA	17336	Broward County FL	50
Anderson County TN	8491	Medina County OH	17090	Wake County NC	17336	Cobb County GA	50
Gibson County TN	8491	Prince Edward County VA	17090	Tuscaloosa County AL	17336	Highlands County FL	50
Sumter County GA	8491	Greenville County SC	17090	Williamson County TN	17336	Alachua County FL	50
Florence County SC	8491	Martin County FL	8545	Gulf County FL	8668	Escambia County FL	50
Marathon County WI	8491	Baker County FL	8545	Jefferson County FL	8668	Lake County FL	50
Towns County GA	8491	Ben Hill County GA	8545	Greene County TN	8668	Richland County SC	50
Frederick County VA	8491	Santa Cruz County CA	8545	Jackson County GA	8668	Volusia County FL	50
Pitt County NC	8491	Rockingham County VA	8545	Madison County MS	8668	Collier County FL	50

Table S4. The optimized evacuation plan (10% increase of transmission rates in destination counties). We show the top 20 destinations for each origin county.

Palm Beach County FL		Broward County FL		Miami-Dade County FL		Monroe County FL	
Leon County FL	67926	Polk County FL	42726	Orange County FL	277387	Dodge County GA	8333
Davidson County TN	25472	Escambia County FL	34181	Hillsborough County FL	60678	St. Lucie County FL	8333
Jefferson County AL	25472	Lake County FL	34181	Osceola County FL	60678	Erie County NY	8333
Marion County IN	16981	Richland County SC	34181	Buncombe County NC	60678	Maricopa County AZ	8333
Jefferson County TN	16981	Volusia County FL	34181	Alachua County FL	34674	Lexington County SC	637
Wake County NC	16981	Collier County FL	34181	Natchitoches Parish LA	26005	Orange County FL	399
Forsyth County GA	12560	Hamilton County TN	34181	Mobile County AL	26005	Fulton County GA	125
Montour County PA	8491	Seminole County FL	34181	Palm Beach County FL	20372	Leon County FL	100
Franklin County VA	8491	Sarasota County FL	25635	Camden County GA	17336	Hillsborough County FL	87
Clay County KY	8491	Sumter County FL	25635	Macon County GA	17336	Osceola County FL	87
Harrison County MS	8491	St. Johns County FL	17090	St. Tammany Parish LA	17336	Buncombe County NC	87
Dougherty County GA	8491	Bay County FL	17090	Forrest County MS	17336	Polk County FL	62
Anderson County TN	8491	Manatee County FL	17090	Lee County AL	17336	Broward County FL	50
Gibson County TN	8491	Medina County OH	17090	Tuscaloosa County AL	17336	Cobb County GA	50
Sumter County GA	8491	Prince Edward County VA	17090	Gulf County FL	8668	Highlands County FL	50
Marathon County WI	8491	Greenville County SC	17090	Jefferson County FL	8668	Alachua County FL	50
Towns County GA	8491	Williamson County TN	17090	Greene County TN	8668	Escambia County FL	50
Frederick County VA	8491	Winston County AL	8545	Jackson County GA	8668	Lake County FL	50
Pitt County NC	8491	Escambia County AL	8545	Madison County MS	8668	Richland County SC	50
Habersham County GA	8491	Robeson County NC	8545	Oneida County WI	8668	Volusia County FL	50

Table S5. The optimized evacuation plan (20% increase of transmission rates in destination counties). We show the top 20 destinations for each origin county.
